# Atomic Imaging of Electrically Switchable Striped Domains in *β*′‐In_2_Se_3_

**DOI:** 10.1002/advs.202100713

**Published:** 2021-07-02

**Authors:** Zhi Chen, Wei Fu, Lin Wang, Wei Yu, Haohan Li, Clement Kok Yong Tan, Ibrahim Abdelwahab, Yan Shao, Chenliang Su, Mingzi Sun, Bolong Huang, Kian Ping Loh

**Affiliations:** ^1^ International Collaborative Laboratory of 2D Materials for Optoelectronics Science and Technology of Ministry of Education Institute of Microscale Optoelectronics College of Chemistry and Environmental Engineering Shenzhen University Shenzhen 518060 China; ^2^ Department of Chemistry National University of Singapore 3 Science Drive 3 Singapore 117543 Singapore; ^3^ Department of Applied Biology and Chemical Technology The Hong Kong Polytechnic University Hung Hom Kowloon Hong Kong SAR China

**Keywords:** antiferroelectrics, ferroelectrics, indium selenide, phase changes, scanning tunneling microscopy

## Abstract

2D ferroelectricity in van‐der‐Waals‐stacked materials such as indium selenide (In_2_Se_3_) has attracted interests because the ferroelectricity is robust even in ultrathin layers, which is useful for the miniaturization of ferroelectric field effect transistors. To implement In_2_Se_3_ in nanoscale ferroelectric devices, an understanding of the domain structure and switching dynamics in the 2D limit is essential. In this study, a biased scanning tunnelling microscopy (STM) tip is used to locally switch polarized domains in *β*′‐In_2_Se_3_, and the reconfiguration of these domains are directly visualized using STM. The room‐temperature surface of *β*′‐In_2_Se_3_ breaks into 1D nanostriped domains, which changes into a zig‐zag striped domains of *β*″ phase at low temperatures. These two types of domains can coexist, and by applying a tip‐sample bias, they can be interchangeably switched locally, showing volatile or nonvolatile like behavior depending on the threshold voltage applied. An atomic model is proposed to explain the switching mechanism based on tip‐induced flexoelectric effect and the ferroelastic switching between *β*′ and *β*″ phases.

## Graphical abstract

With applying tip‐sample bias, field‐driven reversible switching of polarization patterns between antiferroelectric *β*′ and ferroelectric *β*″ phases In_2_Se_3_ are directly visualized in atomic resolution using scanning tunnelling microscopy.

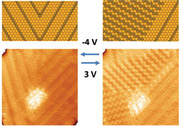

## 1 Introduction

In high‐density nonvolatile memory storage, ultrathin ferroelectric semiconductors are beneficial to resistive switched memory devices owing to their prospect for miniaturisation; in addition, they require only a small voltage for polarization switching compared with thicker films while endowing a higher readout current.^[^ ^1^, ^2^ ^]^ *α*‐In_2_Se_3_ has emerged as a prime candidate for application in highly integrated in‐memory computing.^[^ ^3^, ^4^, ^5^ ^]^ However, the growth of phase‐pure In_2_Se_3_ is challenged by its intricate polymorphism. The easy conversion among *α*, *β*, and *γ* phases during growth renders the phase engineering of the material highly challenging.^[^ ^6^, ^7^, ^8^ ^]^ Slight changes in configurational entropy switches between different polymorphic states result in highly varied crystal symmetries, polarization, and band gaps. Confusion in the phase assignment of *α*‐In_2_Se_3_ and *β*‐In_2_Se_3_ is typical because of the presence of mixed polymorphs in grown or annealed films. Most studies have focused on *α*‐In_2_Se_3_, which has been reported to possess dipole‐locked in‐plane and out‐of‐plane polarization.^[^ ^4^, ^9^, ^10^, ^11^, ^12^, ^13^ ^]^ Although the *β*‐In_2_Se_3_ phase is bulk‐centrosymmetric,^[^ ^14^, ^15^, ^16^ ^]^ the presence of periodic nanostripes on the surface break the inversion symmetry.^[^ ^17^, ^18^ ^]^ Investigating the surface structure of *β*′‐In_2_Se_3_ films is particularly pertinent in view of increasing evidence that it is the most typical phase produced in chemical vapor deposition grown films.^[^ ^19^ ^]^ By analysing the atomic displacement map collected from annular dark‐field transmission electron microscope images, Xu et al. concluded that *β*′‐In_2_Se_3_ is in fact antiferroelectric because the 1D nanostripes are arranged in an antiparallel manner.^[^ ^20^ ^]^ Zhang et al. reported that *β*′‐In_2_Se_3_ transformed into *β*″‐In_2_Se_3_ at a low temperature (77 K), and that the latter exhibited a distinctly different nanostripe pattern compared with that of *β*′‐In_2_Se_3_ (i.e., zig–zag‐like pattern). In addition, their density functional theory (DFT) simulation revealed that *β*″‐In_2_Se_3_ is ferroelectric.^[^ ^19^, ^21^ ^]^ Direct measurement of the electric hysteresis loop on *β*′‐In_2_Se_3_ is technically challenging because of leakage on the highly conducting films; hence, electrical device studies of the *β* phase have revealed only highly conducting properties with no macroscopic evidence of ferroelectricity to clarify the behavior of ferroelectric thin films. Most reports concerning ferroelectricity on In_2_Se_3_ thus far are based on Piezoresponse force microscopy (PFM) studies, which provides a large area aggregate response with little insight into the domain microstructures that determine polarization switching.^[^ ^9^, ^10^, ^11^, ^12^ ^]^

To address the problems above, we performed STM on in situ grown polycrystalline *β*′‐In_2_Se_3_ films, which relax to *β*″‐In_2_Se_3_ at low temperatures. Although the thermally induced phase change between *β*′‐In_2_Se_3_ and *β*″‐In_2_Se_3_ has been studied^19,20^, the electrically switching of domains has not been demonstrated in any beta‐phase In_2_Se_3_ or its derivatives. We discovered that the poly domain structure comprising coexisting *β*′‐In_2_Se_3_ and *β*″‐In_2_Se_3_ can be manipulated at the atomic scale by applying an electric field from an STM tip. Analysis of the atomic configuration in STM images as well as DFT calculations revealed that *β*′‐In_2_Se_3_ is composed of nanostripes arranged in an antiferroelectric manner, whereas *β*″‐In_2_Se_3_ comprises zig–zag nanostripes that contain canted dipoles along a common polar axis. Our study provides the first atomic insights into the complex spatial profile of polarised domains in ultrathin *β*′‐In_2_Se_3_ and their electric field‐induced switching.

## 2 Results and Discussion

**Figure 1:**
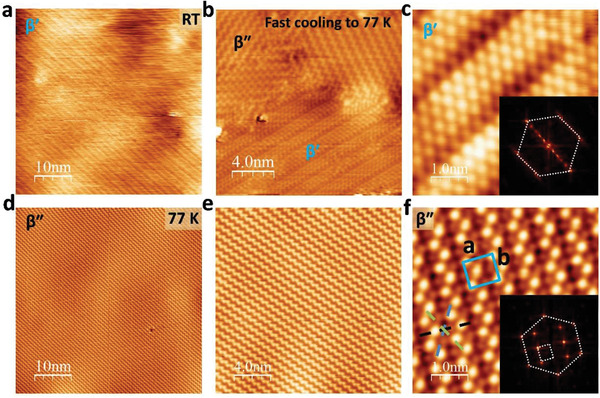
STM images of MBE grown In_2_Se_3_ on HOPG. a) Room temperature STM image of *β*′‐In_2_Se_3_ on HOPG. b) Coexisting *β*′ and *β*″ phases after fast cooling to 77 K. c) Zoomed‐in STM image of *β*′‐In_2_Se_3_ on HOPG after fast cooling from room temperature to 77 K. Inset of (c) shows FFT image of *β*′ phase. d–f) Complete *β*′ to *β*″ conversion after cooling for more than 4 h at 77 K. Inset of (f) shows FFT image *β*″ phase. Dashed white hexagon shows outer ring Se atoms lattice. Dashed white rectangle shows inner rectangular 2 × $\sqrt{3}$ lattice of *β* phase. Scanning bias voltage is a) −0.1 V, b,d,e) 1 V, c) −0.5 V, and f) 0.5 V, respectively. Scanning current for all image is 0.1 nA.

First, we analysed a 1‐nm‐thick In_2_Se_3_ films grown via molecular‐beam epitaxy (MBE). The In_2_Se_3_ film was grown by evaporating In_2_Se_3_ and Se (at a ratio of 1:10) on a highly oriented pyrolytic graphite (HOPG) substrate held at 573 K. The sample was post‐annealed for 1 h at 573 K, followed by cooling to room temperature for STM imaging, whereupon smooth terraces were observed in the large‐area STM image (Figure S1, Supporting Information). The maximum lateral dimension of the atomically flat crystal domain was 500 nm, and the height of one quintuple layer was ≈1.05 nm. **Figure** **1**a shows a room temperature STM image of the sample grown on HOPG, where a topography distinguished by 1D stripes was observed. A zoomed‐in STM image (Figure S1b,c, Supporting Information) show that the stripes were formed by three or four rows of brighter contrast Se atoms alternating with a row of lower contrast Se atoms. These stripes exhibited a three‐fold symmetry approximately the *c*‐axis and a periodicity of 1.6–2.0 nm (Figure 1c). Within the stripe, the Se atoms formed a hexagonal lattice with a unit cell length of 0.40 nm (see inset of Figure 1c). Raman analysis of the as‐grown In_2_Se_3_ revealed phonon signatures of *β*′‐In_2_Se_3_, as judged by the blue‐shifted phonon peaks from the sharp phonon peak of *α*‐In_2_Se_3_ at 104 nm (Figure S2, Supporting Information).

Upon cooling the sample to 180 K, a zig–zag‐shaped *β*″ phase appeared^[^ ^19^ ^]^ and coexisted with *β*′‐In_2_Se_3_ (Figure 1b). Further cooling for another 4 h transformed the *β*′ phase entirely to the *β*″ phase (Figure 1d–f). The *β*″ phase exhibits a basic hexagonal lattice with a lattice constant of 0.40 nm for the surface Se atoms, as shown in the fast Fourier transform (FFT) image (Figure 1f, inset). Furthermore, it contains a rectangular superlattice structure, which can be considered as a 2 × $\sqrt{3}$ reconstruction of the *β* phase (*a* = 7.78 ± 0.20 Å and *b* = 6.93 ± 0.17 Å; blue box in Figure 1f). *β*″‐In_2_Se_3_ indicates a lower formation energy of 0.033 eV per formula unit compared with *β*‐In_2_Se_3_,^[^ ^19^ ^]^ whereas both *β*′‐ and *β*″‐In_2_Se_3_ indicate a lower formation energy compared with *β*‐In_2_Se_3._^[^ ^19^, ^20^ ^]^

To investigate if the substrate affects the *β*′ ↔ *β*″ phase transition, we used MBE to grow *β*′‐In_2_Se_3_ on Au (111), on which large domains were prepared (Figure S3, Supporting Information). After cooling the *β*′‐In_2_Se_3_ on Au(111) (Figure S3a,b, Supporting Information) to 5 K (Figure S3d, Supporting Information), we did not observe a *β*′‐to‐*β*″ phase transition, unlike the case when the film was grown on HOPG. This may be due to the stronger interaction of In_2_Se_3_ with Au(111) than with HOPG, thereby preventing the *β*′ ↔ *β*″ transition. This highlights the importance of the interface strain on polarization switching.

**Figure 2:**
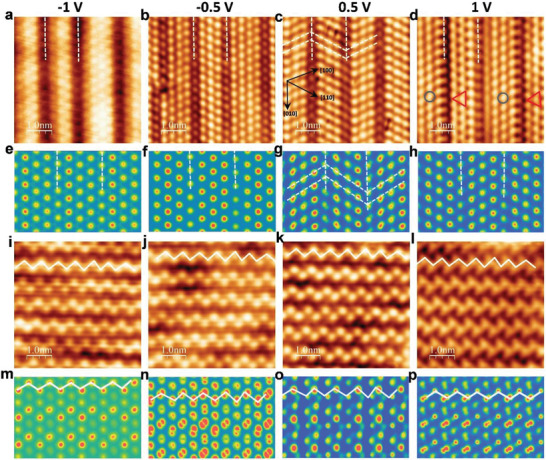
STM and simulated STM images of *β*′‐ and *β*″‐In_2_Se_3_ on Au(111) under different bias voltages. a–d) STM images of *β*′‐In_2_Se_3_ on Au(111) under bias voltages of −1, −0.5, 0.5, and 1 V respectively. Black arrows in (c) indicate [110], [100], and [010] directions. Dashed white lines show zig–zag Se atom rows oriented either along [110] or [100] direction. Triangular‐shaped Se atoms (marked as red triangle in (d)) in one stripe, and round shaped atoms (marked as blue cycle in (d)) in adjacent stripes. e,f) DFT‐simulated STM images of *β*′‐In_2_Se_3_ under bias voltages of −1, −0.5, 0.5, and 1 V respectively. i–l) STM images of *β*″‐In_2_Se_3_ on Au(111) at bias voltages of −1, −0.5, 0.5, and 1 V respectively. m–p) DFT‐simulated STM images of *β*″‐In_2_Se_3_ at bias voltages of −1, −0.5, 0.5, and 1 V respectively. Scanning current for these STM images was set to 0.1 nA.

**Figure 3:**
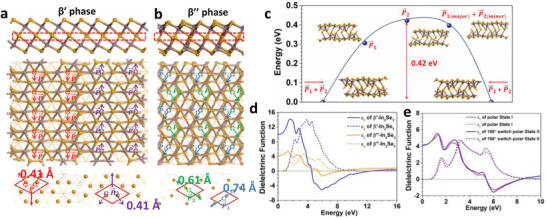
Structure models and dielectric functions of *β*′‐ and *β*″‐In_2_Se_3_. a) Side view (upper panel) and top view (middle and bottom panels) of relaxed structure of *β*′‐In_2_Se_3_. Red and purple arrows differentiate two antiparallel displacements resulting in antiferroelectricity. b) Side view (upper panel) and top view (middle and bottom panel) of relaxed structure of *β*″‐In_2_Se_3_. Blue and green arrows differentiate two in‐plane, 60°‐canted dipoles. c) Transition pathway for polarization switching of *β*″‐In_2_Se_3_. d) Dielectric functions of *β*′‐In_2_Se_3_ and *β*″‐In_2_Se_3_. e) Dielectric function of 180°‐switchable dipoles $\vec{P_{1}}$ and $\vec{P_{2}}$ in *β*″‐In_2_Se_3_.

Bias‐dependent STM images of *β*′ In_2_Se_3_ grown on Au(111) were obtained to determine whether distinguishing electronic features due to contrasting polarity changes appeared. The STM images were simulated based on a DFT‐relaxed structural model of the *β*′ In_2_Se_3_ surface. Details of the simulation are provided in the supporting information. Under negative bias voltages of −1 and −0.5 V, the surface Se atoms of *β*′‐In_2_Se_3_ appeared as bright round dots in the 1D stripes (**Figure** **2**a,b). At 0.5 V, we observed that the Se atom rows oriented either along the [100] or [110] direction in an alternating manner between adjacent stripes (white dashed zig–zag line in Figure 2c). At a bias voltage of 1 V, a zoomed‐in STM image revealed triangle‐shaped Se atoms in one nanostripe (red triangular boxes in Figure 2d) alternating with round‐shaped Se atoms in the adjacent nanostripe (blue cycles in Figure 2d). The simulated STM images (Figure 2e,f) based on the 3 × 3 × 1 *β*′‐In_2_Se_3_ model^[^ ^20^ ^]^ shown in **Figure** **3**a agreed well with the experimental STM images (Figure 2a,b); the relaxed structure of the 3 × 3 × 1 *β*′‐In_2_Se_3_ shows adjacent rows of Se atoms with their centres displaced in opposite directions, resulting in adjacent dipoles oriented in antiparallel directions, i.e., antiferroelectricity. The interaction of the electric field from the tip with different electrostatic charges on adjacent antiferroelectric stripes yielded a slightly different image depicting the electron density around the Se atoms, as shown in Figure 2g,h. Therefore, our STM results are consistent with a previous transmission electron microscopy study of *β*′‐In_2_Se_3_ reporting the antiferroelectric ordering of 1D nanostripes.^[^ ^20^ ^]^ By contrast, the *β*″‐In_2_Se_3_ exhibited a much more disordered structure, where both in‐plane and out‐of‐plane displacements of Se and In atoms were discovered in the DFT relaxed structure (Figure 3b).^[^ ^19^ ^]^ Bias‐dependent STM images of *β*″‐In_2_Se_3_ were obtained at different bias voltages. The experimental (Figure 2i–l) and simulated STM images (Figure 2m–p) show good agreement based on a 3 × 3 × 1 *β*″‐In_2_Se_3_ structure.

In addition, we calculated the macroscopic dielectric response, which is sensitive to the variation in the microscopic structure of the two phases. *β*′‐In_2_Se_3_ exhibits a larger dielectric constant compared with *β*″‐In_2_Se_3_ (Figure 3d). According to our DFT studies, the *β*″‐In_2_Se_3_ phase shows a more distorted structure than *β*′‐In_2_Se_3_ owing to the coupling of polarization to the lattice distortion, which cause both Se and In atoms to displace in the vertical and parallel directions. Two canted dipoles sharing a common polar axis, denoted as $\vec{P_{1}}$ and $\vec{P_{2}}$, were identified; their displacements were 0.74 and 0.61 Å on average, respectively (Figure 3b). Additionally, we calculated the dynamics of the various polarization configurations as the polar axis switched from 0° to 180°. The polarization profile switched between the ground states comprising canted dipoles $\vec{P_{1}}$ and $\vec{P_{2}}$ to intermediate state polarizations aligned predominantly with $\vec{P_{1}}$ or $\vec{P_{2}}$. Two ground states with opposite polarizations comprising canted dipoles of $\vec{P_{1}}$ and $\vec{P_{2}}$ were identified. As shown in Figure 3c, an energy barrier of 0.42 eV was discovered between the ground state and the intermediate state with $\vec{P_{2}}$ polarization. Our calculations confirmed that the dielectric functions of the two ground states with opposite polarizations were identical, indicating that the 180° polarization switching preserved the structural integrity of the ferroelectric phase (Figure 3e).

STM was used to capture the switching of the domain microstructure in time sequence when a tip‐sample voltage pulse was applied. During the image acquisition, scanning was halted momentarily as required by the pulse and then continued immediately. Abrupt changes in the domain structure were visible immediately after the application of a pulse. The presence of a biased STM tip near the surface induced a strong electric field (typically ≥ 10^7 ^V cm^−1^), with the maximum electric field intensity expressed as *E*  ≈  *V* / d_TS_ under the tip apex, where d_TS_ is the tip‐sample spacing, and *V* is the tip‐sample voltage. By applying different voltage pulses, we obtained the statistics of the successful domain switching and identified the threshold voltage required to induce switching. The tip‐sample voltage pulse induced electrostriction effects, where the local dilation or contraction of the lattice volume can occur. Because of the significant strain energy at the boundary between *β*′ and *β*″ domains, strain‐polarization coupling can cause the domains to switch orientations. In our switching experiments, we can broadly classify the domain switch events into (i) the reversible switching of the *β*′ phase from one direction to another, and (ii) the reversible switching of the *β*′ ↔ *β*″ domains. The condition for the coexistence of the thermodynamic equilibrium antiferroelectric and ferroelectric phases is the equality of the thermodynamic potentials of the phases, considering the external and internal effective fields. Because the intrinsic fields were spatially inhomogeneous, at the same value of the external tip‐bias voltage, domain switching occurred only in certain local regions of the sample instead of in the entire sample.

**Figure 4:**
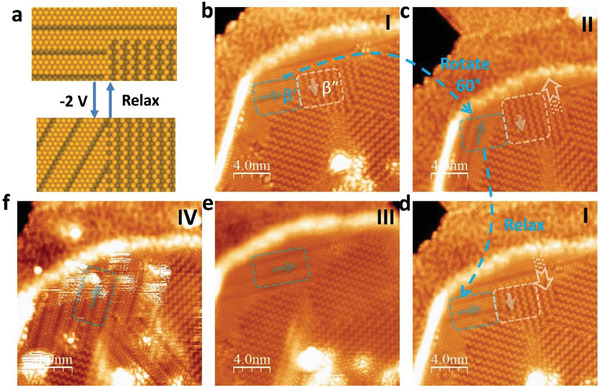
Tip bias‐induced switching of *β*′ by 60° and *β*′ → *β*″ interconversion. a) Schematic illustration showing bias‐induced switching of orientation variants in *β*′ and *β*′ → *β*″ interconversion. b) Configuration “I” showing coexisting *β*′ and *β*″ domains; c) Configuration “I” transformed into configuration “II” after applying −2 V switching bias; *β*′ domain rotates 60° in blue box; *β*″ domains expanded into area of *β*′ domains in white box; d) Configuration “II” relaxed to “I” after 5 min; e) Configuration “III” after applying +6 V switching bias; f) Configuration “IV” after applying −6 V switching bias. Volatile switching of domains occurred at −2 V, whereas nonvolatile switching at ±6 V. All STM images obtained via imaging at *U* = 0.5 V and *I* = 0.1 nA.

A high bias voltage was applied to induce domain switching (writing); subsequently, STM imaging of the domains was performed at a low, nonperturbative voltage of 0.5 V (reading, 0.1 nA). **Figure** **4**b shows state “I”, characterised by coexisting domains of *β*″ and *β*′ phases. The latter was distinguished by thicker looking parallel stripes compared with narrower, zig–zag stripes in *β*″. By applying a −2 V switching bias, state “II” was written, in which the zig–zag stripes of *β*″ domains, as marked in the white box, expanded into the *β*′ phase region (white box and white arrow in Figure 4c). Simultaneously, some *β*′ phases switched their orientation by 60° (blue box and arrow in Figure 4c). The *β*″ phase terminated at an angle of 90° to the *β*′ phase, forming boundaries of elastic and electrostatic discontinuities. If only a low voltage of −2 V is used for switching, then state “II” will appear as “volatile” and hence relaxes back to the original state “I” in ≈5 min (Figure 4d), indicative of the shape memory effect. However, if a higher voltage of +6 V is used to switch the domain, then a more permanent state “III” is created (i.e., nonvolatile) (Figure 4e). The *β*′ domain can be switched back to 60° using a reverse voltage of −6 V, and state “IV” is stable and does not relax back to state “III” (Figure 4e).

The *β*′ phase shows three structurally equivalent orientation variants that can be switched from one to another by applying a tip‐sample bias. Both *β*′ and *β*″‐In_2_Se_3_ are characterised by three orientation variants because of the three‐fold symmetry, and ferroelastic‐type transitions are possible between them. These nanostripes are reminiscent of the low‐symmetry distorted crystal structure in 1T MoTe_2_, which exhibits a three‐fold orientation variation in its domain direction, and where a small strain can cause the switching of domain orientation.^[^ ^22^ ^]^

**Figure 5:**
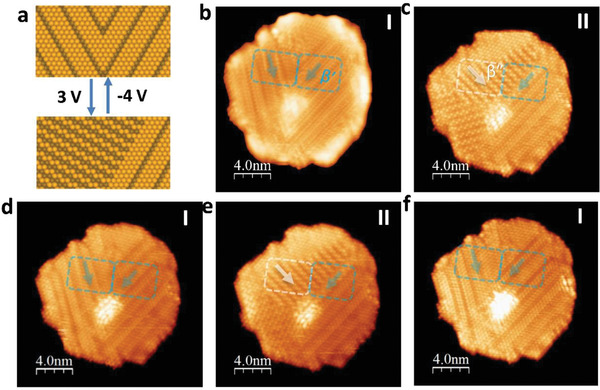
Electron‐injection‐induced switching in *β*′ ↔ *β*″ phase transformation. a) Schematic illustration showing bias‐induced rotation‐transformations of *β*′ and *β*″ domains switched by electrical biases of +3 and −4 V. b) Configuration “I” showing two domains of *β*′‐In_2_Se_3_; c) Configuration “I” transformed into configuration “II” after applying +3 V switching bias; d) Configuration “II” reverted to “I” after applying −4 V switching bias; e) “I” switched back to “II” after applying +3 V switching bias; f) Configuration “II” reverted to “I” after applying −4 V switching bias. All STM images obtained via imaging at *U* = 0.5 V and *I* = 0.1 nA.

In some regions of the *β*′ domain, applying a bias voltage of +3 V switched the *β*′ phase to the *β*″ phase. As shown in **Figure** **5**b,c, the striped *β*′ phase and zig–zag *β*″ were oriented at either 30° or 90° to each other. The *β*″ phase can be switched back to the *β*′ phase by applying a −4 V bias voltage (Figure 5f). This *β*″ ↔ *β*′ phase transformation can be cycled repeatedly (Figure 5e,f). The orientation switching of the *β*″ domain by 60° can occur as well; however, this typically requires a higher switching voltage (6–8 V) (Figure S4, Supporting Information) than that in the *β*′ domain. As shown in both Figures 4 and 5, it is noteworthy that the switching of the *β*″ domain occurs simultaneously with the extension of the *β*′ domain boundaries into *β*″, suggesting a cooperative mechanism whereby the *β*′ domains provide nucleation sites for newly formed *β*″ domains.

**Figure 6:**
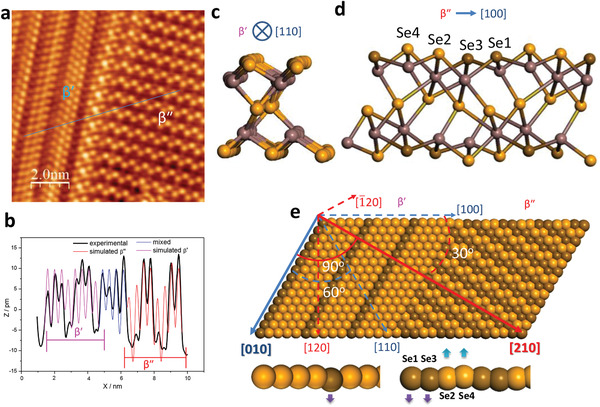
Proposed model of *β* ↔ *β*′ phase conversion by atomic reconfiguration. a) High‐resolution STM image of coexisting *β*′ and *β*″ phases oriented 90° with each other. b) Height profile traced by blue line in (a), plotted together with DFT‐simulated height profiles of *β*′ and *β*″ phases. c) Structure of *β*′ phase from DFT calculation.^[^ ^20^ ^]^ d) Structure of *β*″ phase from DFT calculation.^[^ ^19^ ^]^ e) Proposed model of *β*′ ↔ *β*″ phase conversion by atomic reconfiguration. Scanning parameter for (a): *U* = 0.5 V; *I* = 0.1 nA.

How does the *β*′ phase convert into *β*″? Is the crossing of the interphase domain wall (from one phase to the other) accompanied by the continuous conjugation of the crystal planes (free of breaks and dislocations), or does it involve dislocations or intermediate phase? Figure 1b shows a close‐up STM image of the dislocation‐free boundaries between the *β*′ and *β*″ phases, which intersected at either 30° or 90°. Based on structural analysis, we speculate that the atomic reconfiguration of the *β*′ phase to the *β*″ phase can be accomplished in a continuous conjugation manner by the vertical displacement of Se atoms. **Figure** **6**a shows a magnified STM image of atoms at the 90° junction during the *β*′ → *β*″ transition. A schematic illustration depicting the manner by which the *β*′ phase converts to the *β*″ phase through the collective displacement of atom pairs along the [100] direction is shown in Figure 6e. The three‐fold symmetric *β*′ phase contained stripes in the <100> directions. In response to a tip‐sample bias voltage, the alternating atom pairs along the [100] direction became vertically displaced (lower half of Figure 6e). Adjacent rows of these corrugated atom pairs (orange or brown) zig–zagged along the [210] direction and transformed into *β*″ stripes. As shown in Figure 6a, because the *β*″ phase was derived from the *β*′ phase, the zig–zag *β*′ and *β*″ stripes intersected at 30° or 90° along their long axes, as observed in the domain switching experiments (see Figure 5). The DFT optimised structure of the *β*″ phase^[^ ^19^ ^]^ shows that Se atom pairs 2 and 4 as well as Se atom pairs 1 and 3 were alternatively displaced outwardly and inwardly with respect to each other (Figure 6d). STM it is sensitive to the vertical displacement of surface atoms that accompanied the elastic deformation, the STM height profile of the two phases shown in Figure 6b confirmed that the *β*″ phase exhibited a larger vertical corrugation compared with the *β*′ phase.

## 3 Conclusion

We have observed the dynamic reconfiguration of nanosized domains in *β*′‐In_2_Se_3_. Dipole ordering in an inhomogeneous two‐phase state can be switched using a tip‐sample bias. We observed that the domain movements of *β*′‐In_2_Se_3_ and *β*″‐In_2_Se_3_ phases were coupled by elastic strain during electrical switching, and that *β*′ ↔ *β*″ interconversion involved a 30° or 90° swing of the 1D domains. Switching between orientation variants of the *β*′ or *β*″ phase occurred by a 60° swing. Hence, our study provides the first atomic insights into the mechanism of the electrically switching of *β*′ and *β*″‐In_2_Se_3_ domains in In_2_Se_3_. Furthermore, our results would benefit the emerging paradigm of domain‐wall nanoelectronics.

## 4 Experimental Section

Experiments were performed in an ultrahigh vacuum system (pressure < 2.0 × 10^−10^ mbar) equipped with a Unisoku USM1300 low‐temperature scanning tunnelling microscope. STM was performed under the constant‐current mode and a liquid nitrogen temperature of 77 K. STM data were analysed and rendered using WSxM software. Indium selenide vapor was generated by evaporating In_2_Se_3_ granules (purity 99.99%, Alfa‐Aesar) heated in a Mo crucible inside a K‐cell at a temperature of 1023 K, whereas selenium vapor was generated using a selenium evaporation cell. In_2_Se_3_ layers were grown on both HOPG and clean Au(111) substrates. The Au(111) single crystal was cleaned via repeated cycles of Ar^+^ sputtering (1.0 keV), followed by annealing at 873 K.

## Conflict of Interest

The authors declare no conflict of interest.

## Author Contributions

Z.C. and K.P.L. conceived and designed the experiments. Z.C., W.F., and C.K.Y.T. synthesized In_2_Se_3_ samples by MBE method. Z.C., Y.S., and H.H.L. performed the STM measurements. L.W. and W.Y. performed the Raman measurements and take part in the discussion. I.A. performed the SHG measurement. B.H. carried out the theoretical calculations., S.M. assisted B.H. in analysis. Z.C. and K.P.L. analyzed the data and wrote the paper with input from all the authors.

## Supporting information

Supporting Information

## Data Availability

Data is available on request from the authors. The data that support the findings of this study are available from the corresponding author upon reasonable request.
