# Supplementary material for: Atomic Imaging of Electrically Switchable Striped Domains in β′‐In2Se3
Source: Adv Sci (Weinh). 2021 Jul 2;8(17):2100713. doi: 10.1002/advs.202100713 (PMC8425890; doi:10.1002/advs.202100713)
Supplement: Supplementary file 1 — Supporting Information [file ADVS-8-2100713-s001.pdf]

© 2021 Wiley-VCH GmbH

ADVANCED SCIENCE
Open Access

Supporting Information

for *Adv. Sci.,* DOI: 10.1002/advs.202100713

Atomic Imaging of Electrically Switchable Striped Domains in β′-In_2Se_3

Zhi Chen, Wei Fu, Lin Wang, Wei Yu, Haohan Li, Clement Kok Yong Tan, Ibrahim Abdelwahab, Yan Shao, Chenliang Su, Mingzi Sun, Bolong Huang, and Kian Ping Loh**

Supporting Information for

Atomic Imaging of Electrically Switchable Striped Domains in β′-In_2Se_3

Zhi Chen[1,2], Wei Fu[2], Lin Wang[2], Wei Yu[2], Haohan Li[2], Clement Kok Yong Tan[2], Ibrahim Abdelwahab[2], Yan Shao[2], Chenliang Su[1], Mingzi Sun[3], Bolong Huang[3]*, Kian Ping Loh[1,2]*

[1]International Collaborative Laboratory of 2D Materials for Optoelectronics Science and Technology of Ministry of Education, Institute of Microscale Optoelectronics, College of Chemistry and Environmental Engineering. Shenzhen University, Shenzhen, 518060, China.
[2]Department of Chemistry, National University of Singapore, 3 Science Drive 3, Singapore 117543, Singapore.
[3]Department of Applied Biology and Chemical Technology, The Hong Kong Polytechnic University, Hung Hom, Kowloon, Hong Kong SAR, China.
*e-mail: chmlohkp@nus.edu.sg; bhuang@polyu.edu.hk

Calculation detail:
We have applied the CASTEP package for all the theoretical calculations in this work.[1] For all the calculations, we have applied the GGA within PBE to describe the exchange-correlation energy.[2,3] The ultrasoft pseudopotential with cut-off energy of 380 eV is applied. For all the geometry optimization, the Broyden-Fletcher-Goldfarb-Shannon (BFGS) algorithm is selected to converge the Hellmann-Feynman forces to less than 0.001 eV/Å.[4] Considering the DFT computational cost, the Monkhost-Pack reciprocal space integration was performed using coarse k-points with a mesh of 2 × 2 × 1,[5] which was guided by the initial convergence test. The ultra-fine convergence criteria are set based on the tolerances of the total energy and the inter-ionic displacement should not exceed 5 × 10^{-6} eV per atom and 5 × 10^{-4} Å per atom, respectively.

For the simulation of STM, the 3 × 3 × 1 single layer β′ and β″ In_2Se_3 have been chosen as the model in this work. To be consistent with the experimental results, we have applied the bias voltage of -1.0 V, -0.5 V, 0.5 V and 1.0 V, respectively, to simulate the STM results. The STM results have been represented by an isosurface of the electron density generated only by states at a certain energy away from the Fermi level. The distance from the Fermi level corresponds to the applied bias in STM experiments: positive bias corresponds to empty (conduction) states and negative bias to occupied (valence) states. This approach neglects the actual geometry of the STM tip.

For STM simulation, the Bardeen approximation of tunnelling current expressions is shown below.

$$I = \frac{4\pi e}{\hbar}\int_0^{eV} \rho_{\mathrm{s}}(E_{\mathrm{F}} - eV + \varepsilon)\,\rho_{\mathrm{t}}(E_{\mathrm{F}} + \varepsilon)|M|^2 d\varepsilon$$

eV represents the bias voltage and $|M|^2$ is the tunnelling matrix element. ρ_{t} and ρ_{s} are the state's density of tip and sample, respectively. Since ρ_{t} and are close to constant, which leads the direct revel of state density of sample by dI/dV spectra, enabling the direct comparison between the STS results and the TDOS of theoretical calculations as well.

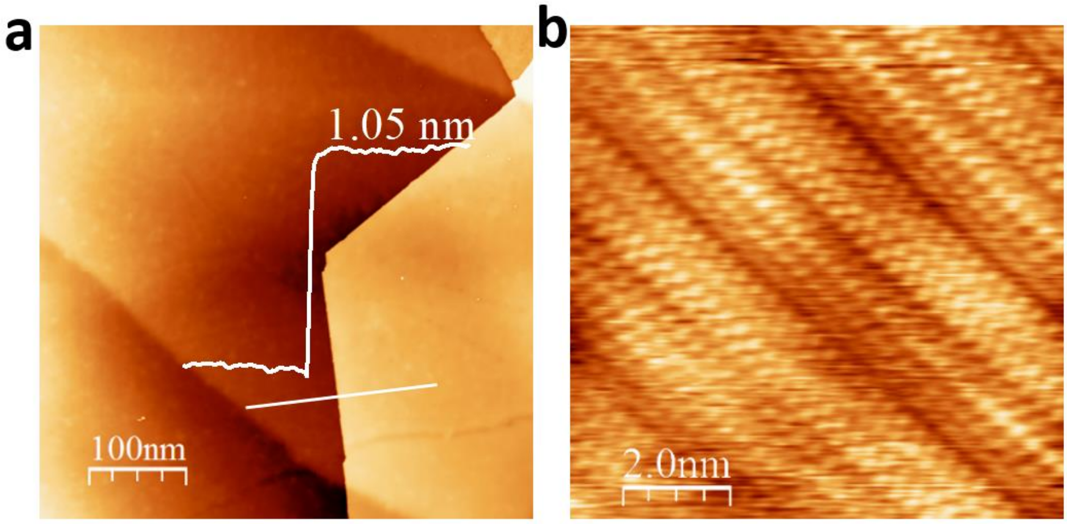

Figure S1. STM images of MBE grown In_2Se_3 on HOPG. a) Large scale of In_2Se_3 on HOPG. b) Zoom-in STM image of β′-In_2Se_3 on HOPG at room temperature. Scanning parameter for (a) U = 2 V, I = 0.1 nA; (b) U = -0.2 V, I = 0.1 nA.

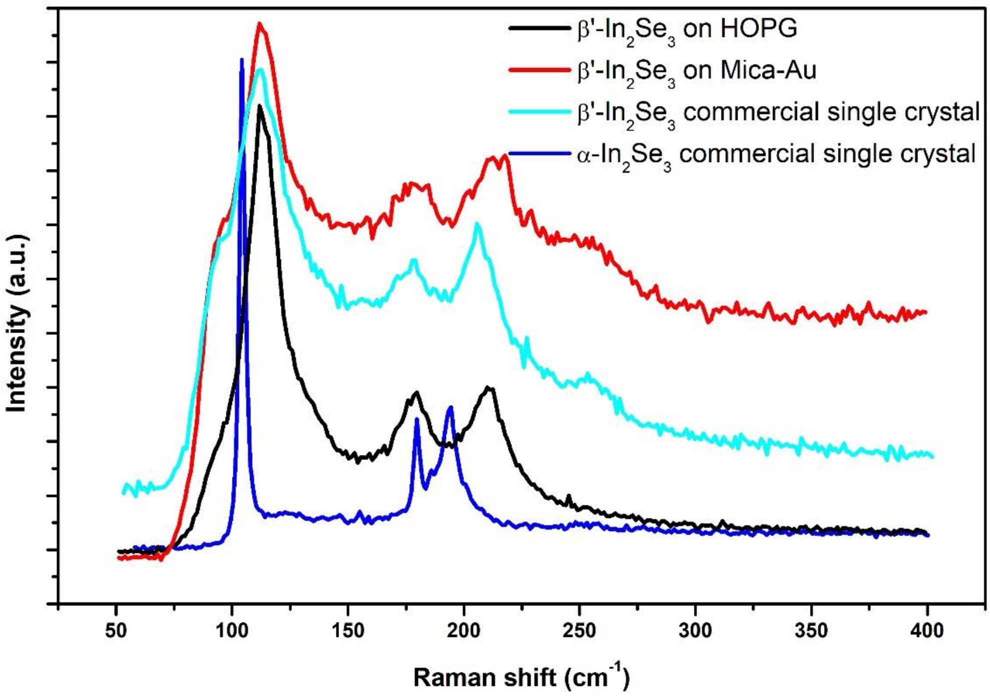

Figure S2. Raman spectra of β′-In_2Se_3 grown on HOPG and Au(111), and commercial α- and β′-In_2Se_3 single crystals.

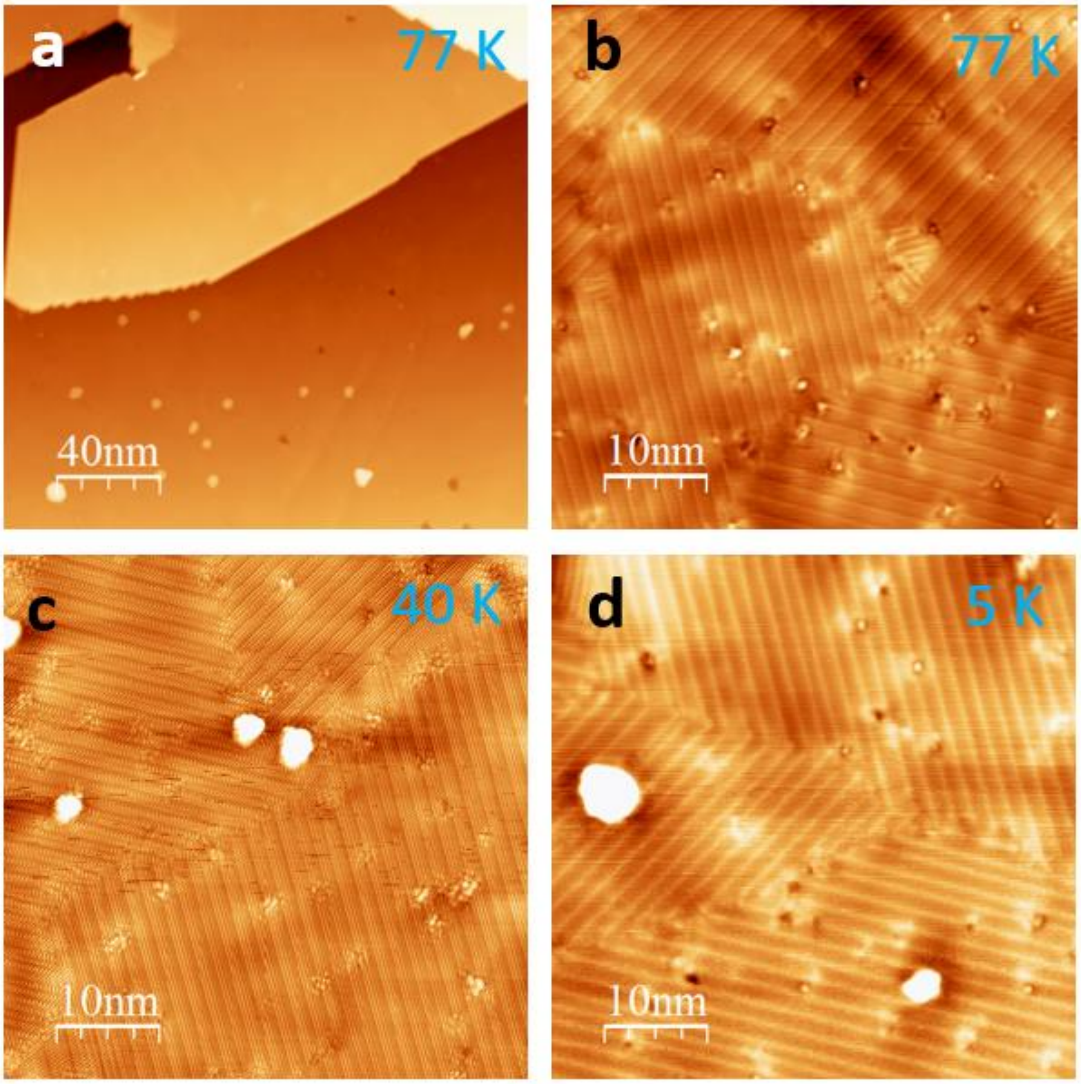

Figure S3. STM images of β′-In_2Se_3 recorded on Au(111) at different temperature. The MBE grown In_2Se_3 which was deposited 30 min on Au(111) held at 573 K. The scanning temperature are (a,b) 77 K, (c) 40 K and (d) 5 K, respectively. Scanning parameter for (a-d): U = 1 V, I = 0.1 nA.

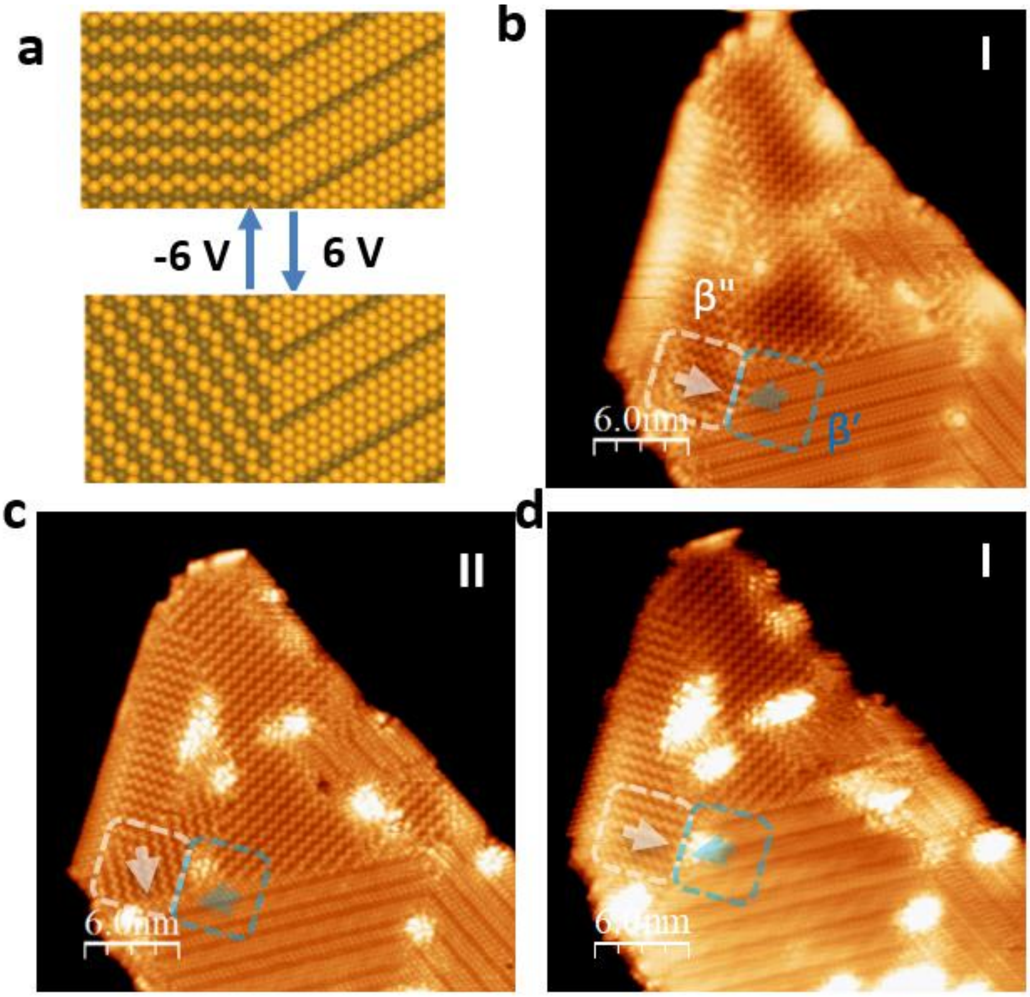

Figure S4. Bias-induced rotation of β″ phase domain orientation**.** a) Schematic showing the bias-induced switching of orientation variants in β″ phase. b) Configuration "I" showing co-existing β′ and β″ domains. c) Configuration "I" changes into configuration "II" after applying a switching bias of +6 V. The β″ domain in white box rotates 60°. d) configuration "I" was obtained after switching bias of -6 V. All STM images collected at U = 0.5 V, I = 0.1 nA.

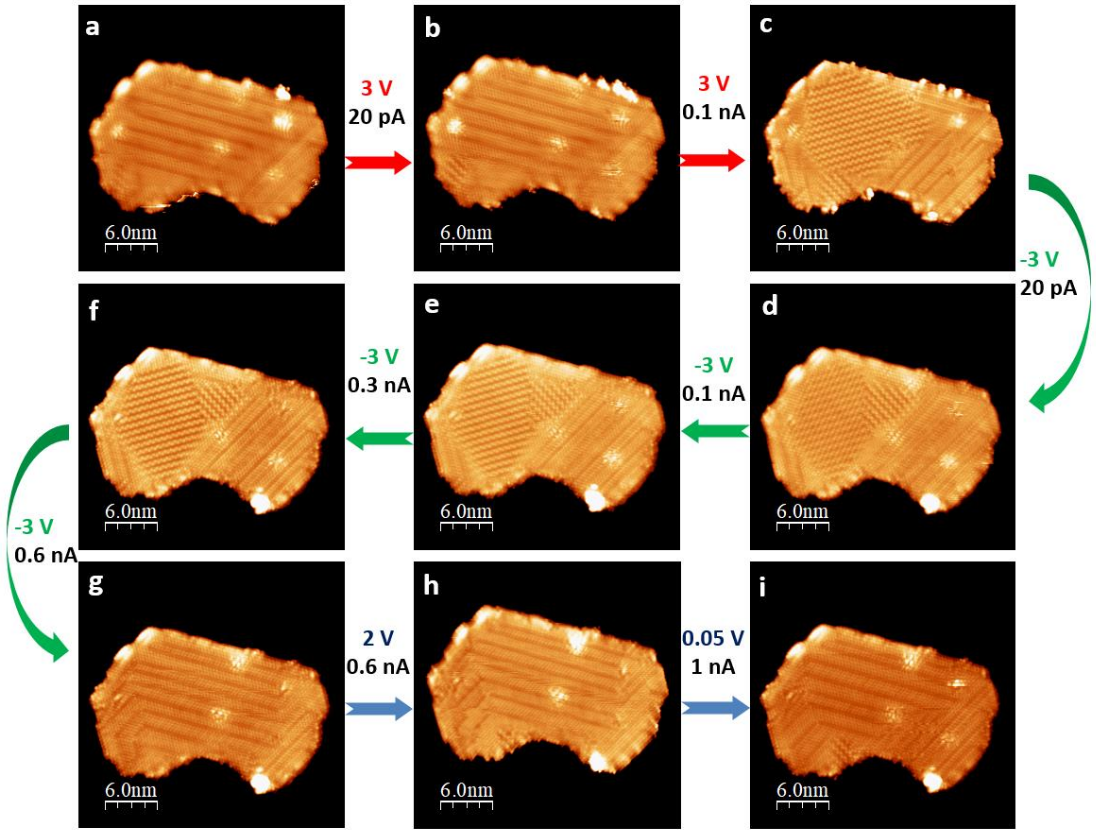

Figure S5. Current affects the β′↔β″ phase transition. a) STM image shows a β′ phase at 0.5 V. b) When a 3 V, 20 pA current is applied, β′→β″ phase transformation does not occur. c) When a 3 V, 0.1 nA current is applied, β′→β″ phase transformation occurs. Application of -3 V and 20 pA (d), -3 V and 0.1 nA (e), -3 V and 0.3 nA (f) does not switch β″ back to β′ phase. g) Further increasing current to 0.6 nA, the phase transition of β″→β′ occurs. However, when further closer the tip to the surface by lower the voltage and increase the current (2 V, 0.6 nA (h) and 0.05 V, 1 nA (i)), does not make the phase transition occurs. All STM images collected by imaging at U = 0.5 V, I = 0.1 nA. These results mean this kind of phase transition only occurs when bias is up to certain value. When up to the bias, increasing the current can also help the phase transition.

Reference:

[1] S. J. Clark, M. D. Segall, C. J. Pickard, P. J. Hasnip, M. I. J. Probert, K. Refson, M. C. Payne, *zkri* **2005**, *220*, 567.

[2] J. P. Perdew, K. Burke, M. Ernzerhof, *Phys. Rev. Lett.* **1996**, *77*, 3865-3868.

[3] J. P. Perdew, J. A. Chevary, S. H. Vosko, K. A. Jackson, M. R. Pederson, D. J. Singh, C. Fiolhais, *Phys. Rev. B* **1992**, *46*, 6671-6687.

[4] J. D. Head, M. C. Zerner, *Chem. Phys. Lett.* **1985**, *122*, 264-270.

[5] M. I. J. Probert, M. C. Payne, *Phys Rev B* **2003**, *67*, 075204.
